# Non-Coding RNAs in the Crosstalk between Breast Cancer Cells and Tumor-Associated Macrophages

**DOI:** 10.3390/ncrna8010016

**Published:** 2022-02-06

**Authors:** Anna Benedetti, Chiara Turco, Giulia Fontemaggi, Francesco Fazi

**Affiliations:** 1 Section of Histology and Medical Embryology, Department of Anatomical, Histological, Forensic and Orthopedic Sciences, Sapienza University of Rome, 00161 Rome, Italy; anna.benedetti@uniroma1.it; 2 Oncogenomic and Epigenetic Unit, IRCCS Regina Elena National Cancer Institute-IFO, 00144 Rome, Italy; chiara.turco@ifo.gov.it (C.T.); giulia.fontemaggi@ifo.gov.it (G.F.)

**Keywords:** tumor-associated macrophages, microRNA, long non-coding RNA, breast cancer

## Abstract

Non-coding RNAs (ncRNAs) play a pivotal role in regulating the tumor microenvironment (TME) by controlling gene expression at multiple levels. In tumors, ncRNAs can mediate the crosstalk between cancer cells and other cells in the TME, such as immune cells, stromal cells, and endothelial cells, influencing tumor development and progression. Tumor-associated macrophages (TAMs) are among the most abundant inflammatory cells infiltrating solid cancers that promote tumorigenesis, and their infiltration correlates with a poor prognosis in many tumors. Cancer cells produce different ncRNAs that orchestrate TAM recruitment and polarization toward a tumor-promoting phenotype. Tumor-reprogrammed macrophages shape the TME by promoting angiogenesis and tissue remodeling, and suppressing the anti-tumor activity of adaptive immune cells. TAMs can also produce ncRNA molecules that boost cancer cell proliferation and direct their phenotype and metabolic changes facilitating cancer progression and metastasis. This review will focus on the crosstalk between cancer cells and TAMs mediated by microRNAs and long non-coding RNAs during breast cancer (BC) initiation and progression.

## 1. Introduction

The communication of cancer cells with the surrounding environment is crucial for tumor development and progression. Cancer cells direct the formation of a TME that sustains their nutrient demand by spreading through the blood to other organs, and the escape from immune surveillance [1,2,3]. The TME includes cancer cells, blood vessels, immune cells, and stromal cells with the adjacent extracellular matrix (ECM) [3,4]. TAMs represent one of the primary infiltrating myeloid cells that are part of the TME, and their presence correlates with tumor aggressiveness and bad prognosis in breast cancer and other tumors [5].

TAMs partly originate from tissue-resident macrophages, but they mostly derive from circulating monocytic precursors recruited to the tumor site by chemotactic signals released by cancer cells [6]. These chemotactic signals include cytokines such as CSF-1, and chemokines such as CCL-2 and CCL-5, which recruit inflammatory monocytes and direct TAM differentiation [6,7]. Although TAMs can support or inhibit cancer progression depending on their activation state and polarization, many studies have shown that they mostly polarize toward a tumor-promoting phenotype [6,7]. Macrophages can differentiate into the pro-inflammatory M1 phenotype or the anti-inflammatory M2 phenotype [8]. Classical M1 macrophages are driven by IFNγ and bacteria during TH1 (type I helper) responses against pathogens, and produce inflammatory cytokines and tissue-damaging factors [9]. M1-like macrophages exert an anti-tumor activity during tumor onset by distinguishing and killing malignant cells. However, in established tumors, cancer cells drive macrophage shift into the alternative M2 phenotype [6,10]. M2 macrophages mediate tissue repair and remodeling after inflammatory responses in healthy tissues [11]. However, in the TME, macrophages promote cancer cell proliferation, epithelial-to-mesenchymal transformation (EMT), and stemness [12]. Moreover, TAMs favor metastasis formation by inducing angiogenesis and tissue remodeling, and contribute to the generation of an immunosuppressive environment that protects cancer cells from being killed by other immune cells [7,13].

Many studies have extensively identified macrophages as CD68-expressing cells in tumor tissues. The M2-like population is commonly distinguished for the expression of CD163, CD204, and CD206; on the contrary, M1-like macrophages express HLA-DR, iNOS, and pSTAT1, although these markers alone are not exclusive for M1 macrophages [10].

Classical M1 and alternative M2 macrophages are only two extremes of a broad spectrum of possible phenotypes. In tumors, the multitude of signals from the TME leads to TAM phenotypic plasticity and heterogeneous functions ranging between M1 and M2 phenotypes [6,14].

In recent years, many studies have shown that non-coding RNAs’ (ncRNAs) biosynthesis and processing are significantly altered in malignant cells, contributing to atypical cancer cell behavior [15,16].

Non-coding RNAs are a class of RNAs that lack a protein-coding region but can influence gene expression in healthy and pathological contexts. NcRNAs include small ncRNAs shorter than 200 nucleotides (nt) and long ncRNAs (lncRNAs) longer than 200 nt. Shorter RNAs can be further divided into small nucleolar RNAs (snoRNAs), small interfering RNAs (siRNAs), small nuclear RNAs (snRNAs), transfer RNAs (tRNAs), Piwi-interacting RNAs (piRNAs), and the most studied microRNAs (miRNAs) [17]. miRNAs can regulate post-transcriptional gene expression by repressing or degrading complementary mRNAs, and originate from a long primary miRNA (pri-miRNA) processed by the nuclear ribonuclease Drosha to a long precursor miRNA (pre-miRNA). Finally, the pre-miRNA is exported from the nucleus and cleaved by the RNase enzyme Dicer to produce the mature double-stranded miRNA [17,18]. The active single-stranded miRNA is then loaded into the RISC (RNA-induced silencing complex), where a member of the Argonaute (Ago) family of proteins allows its functional activity [19,20].

LncRNAs biosynthesis is similar to that of miRNAs for many aspects. LncRNAs can control gene expression by directly interacting with proteins, lipids, DNA, and RNA. However, because of their ability to bind to different molecules, lncRNAs functions are more complex than that of miRNAs. They can regulate many cellular processes, such as translation, splicing, and chromatin status, by interacting with RNA/DNA or influencing transcription factor stability and localization [21,22,23].

NcRNAs influence different aspects of tumor onset and development by promoting or suppressing tumorigenesis or affecting drug resistance. Moreover, the release of ncRNAs inside exosomes mediates the communication between cancer cells and other cells in the TME [24]. Researchers have identified several miRNAs in breast cancer that regulate the expression levels of ERα [25], cell survival and apoptosis [26], EMT [27], and cell stemness [28]. Like miRNAs, lncRNAs can be up-downregulated or differentially expressed in the various breast cancer subtypes and drive breast cancer progression by regulating pro-metastatic factors [29]. This review will focus on the miRNAs and the lncRNAs involved in breast cancer cell-TAM crosstalk.

## 2. Role of Non-Coding RNAs in TAM Recruitment and Polarization in Breast Cancer

Non-coding RNAs produced by cancer cells may regulate monocyte recruitment to the tumor site and direct their differentiation into M1 or M2 macrophages by influencing the expression of pro-or anti-inflammatory genes in macrophages [30,31].

Monocyte recruitment is guided by chemotactic signals released by cancer cells. One of the most potent factors involved in this process is CCL2, upregulated in many cancers [32,33]. Recently, Frank et al. found that miR-375 induces CCL2 expression in breast cancer cells [34]. MiR-375 is released by apoptotic BC cells and stimulates the production of CCL2 in other cancer cells by unknown mechanisms. Furthermore, miR-375 binds to the macrophage CD36 receptor and is transferred to TAMs, where it directly binds and downregulates TNS3 and PXN, two migration-inhibitory proteins, increasing the macrophage’s ability to infiltrate the tumor, resulting in cancer progression [34]. CCL2 mediates macrophage recruitment also at metastatic sites [35]. LncRNA associated with breast cancer brain metastases (lnc-BM) is significantly upregulated in brain metastasis and influences TAM recruitment by inducing the production of CCL2 via JAK2/STAT3 pathway activation in cancer cells [36]. The cytokine colony-stimulating factor-1 (CSF1) stimulates macrophage recruitment and survival, and its expression is prognostic for mortality in breast cancer [37,38]. Mir-149 directly targets and inhibits CSF1 mRNA in breast cancer cells, limiting macrophage recruitment to the primary tumor. The reduced TAM infiltration has also been associated with decreased lung metastasis formation in a mouse model with orthotopically injected MDA-MB-231 cells [39]. Once in the tumor site, monocytes rapidly differentiate and are polarized into M1 or M2-like macrophages, depending on the stimuli from the TME [6,7]. MiR-149 suppresses the expression of two M2 markers in macrophages, the mannose receptor C-type 1 (MRC1) and arginase-1 (ARG1). Although the authors did not investigate the mechanism, miR-149 possibly acts in a paracrine manner by regulating the production of soluble factors secreted by BC cells that influence macrophage polarization [39].

The miR-200 tumor suppressor family are well-known negative regulators of Epithelial-mesenchymal transition (EMT), and their expression is downregulated in many aggressive cancers, including breast cancer [40]. Williams et al. reported that the restoration of miR-200c expression in BC cells stimulates GM-CSF secretion, which induces M1-macrophage polarization as demonstrated by the upregulation of the M1 marker Nos2 and the downregulation of the M2 marker Arg1 [41]. Indeed, GM-CSF is an important regulator of the pro-inflammatory M1 phenotype [42]. On the contrary, other studies have shown that miR-200 expression is associated with poor prognosis in breast cancer patients [43,44]. Accordingly, Meng et al. reported that miR-200c upregulates PAI-2 expression in BC cells and promotes the secretion of the anti-inflammatory cytokine IL-10, which directly stimulates M2-like polarization [45].

Several cell types present in the TME, such as cancer cells, fibroblasts, and immune cells, produce a high level of IL-6 cytokine, which correlates with low survival rates in breast cancer [46]. IL-6 produced by breast cancer cells can induce M2-macrophage polarization by leading to the inhibition of miR-19a-3p expression in TAMs. Mechanistically, the downregulation of miR-19a-3p releases its inhibitory activity on the Fra1 gene, activating the downstream STAT3 pathway which drives M2-polarization [47].

Wang et al. reported that miR-100 is highly expressed in TAMs from breast cancer and in the surrounding cancer tissue. High expression of miR-100 in TAMs favors M2 TAM polarization by inhibiting the mTOR pathway and leading to the upregulation of the M2 marker CD206 [48].

Tao et al. showed that the expression of the lncRNA linc00514 in breast cancer tissues correlates with tumor aggressiveness and controls TAM polarization via Notch signaling activation [49]. In BC cells, linc00514 induces a STAT3-mediated increase in Jagged1, promoting the secretion of the anti-inflammatory cytokines IL-4 and IL-6, which facilitate M2 polarization by increasing the expression of the M2 markers CD206 and CD163 in TAMs [49].

Xist is a tumor-suppressor and poorly expresses lncRNA in BC cells [50]. Recently, Zhao et al. showed that Xist is also expressed in macrophages, and it participates in M1-phenotype maintenance. Xist functions as a sponge for miR-101, which inhibits the expression of C/EBPα and KLF6, two regulators of M1 macrophage polarization. The knockdown of Xist in M1 macrophages promotes the switch to the M2 phenotype, as demonstrated by the upregulation of the M2 marker CD206, confirming its tumor-suppressor role not only in BC cells but also in TAMs [51]. Several studies have reported the oncogenic function of miR-181 in breast cancer [52]. MiR-181 can be released into BC cell-derived exosomes, and promotes the generation of an immunosuppressive environment by enhancing the infiltration of myeloid-derived suppressor cells, which play a crucial role in cancer immune escape [53]. In TAMs, miR-181a suppresses the expression of C/EBPα and KLF6, promoting M2-polarization and increasing TAM pro-tumoral behavior [54].

The lncRNA GNAS-AS1 is upregulated in ER+ breast cancer tissues and TAMs from BC tissues. The overexpression of GNAS-AS1 facilitates M2 macrophage polarization in vitro by directly sponging miR-433-3p, a negative regulator of GATA3, which induces M2-macrophage polarization and promotes anti-inflammatory activity [55]. GATA3 is also targeted and inhibited by miR-720, an anti-oncogenic miRNA downregulated in TAMs from metastatic breast cancer patients. The restoration of miR-720 expression in M2-macrophages drastically reduces the production of anti-inflammatory cytokines and the ability of macrophages to stimulate BC cell migration [56].

**Figure 1:**
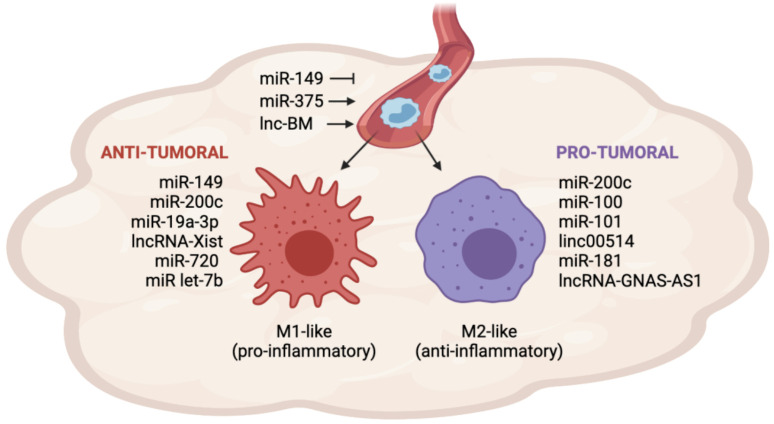
miRNAs and lncRNAs involved in TAM recruitment and polarization in breast cancer. The expression of miRNAs and lncRNAs in BC cells influences TAM recruitment by regulating the secretion of cytokines and chemokines in the TME. MiR-149 inhibits monocyte recruitment, whereas miR-375 and lnc-BM promote monocyte recruitment. Once in the tumor site, monocytes can differentiate into anti-tumoral (M1-like) or pro-tumoral (M2-like) macrophages. The expression of miR-200c, miR-100, miR-101, linc00514, miR-181 and lncRNA GNAS-AS1 in BC cells or TAMs promotes the differentiation into the M2-like phenotype. Conversely, the expression of miR-149, miR-200c, miR-19a-3p, lncRNA-Xist, miR-720, and miR-let7b in BC cells or TAMs promotes the differentiation into the M1-like phenotype.

NcRNAs have also been studied as drugs to re-educate TAMs in vivo [57]. Huang et al. constructed a nanoparticle loaded with miRNA mimic let-7b, which can bind to the TLR7 on TAM membrane. In vitro, let-7b acts as an agonist of TLR7 and inhibits the secretion of IL-10, a potent immunosuppressive cytokine that induces M2-like polarization. Once delivered in vivo in a mouse model of breast cancer, the let-7b-complex is released in the tumor acidic microenvironment and limits tumor growth by reversing M2-like TAM phenotypes. Moreover, let-7b can also activate the TAM-induced Th1 response in the treated mice, restoring the anti-tumor immune response [58]. Figure 1 and Table 1 and Table 2 recapitulate the role of miRNAs and lncRNAs in TAM recruitment and polarization in breast cancer.

## 3. Non-Coding RNAs in the Regulation of BC Cells and TAM Metabolic Reprogramming

Cancer cells profoundly alter their metabolism to cope with the high biosynthetic demand of proliferating cells and adopt aerobic glycolysis, also known as the Warburg effect, to enhance glucose uptake and utilization [81]. In aerobic glycolysis, glucose is processed into lactate even in the presence of oxygen, providing ATP and building blocks very quickly to support tumor cell survival and proliferation [81,82]. In this context, TAM metabolism is also reprogrammed to sustain the newly developed tumor microenvironment [83,84]. Despite TAM phenotype changes over time during tumor progression, M1-like macrophages generally display a highly glycolytic metabolism associated with lactate secretion and ROS production. Instead, M2-like macrophages typically rely on oxidative phosphorylation and fatty acid oxidation [83,84]. Cancer cells and TAMs establish a mutual metabolic communication, with cancer cells promoting the M2-like phenotype through the secretion of lactate, and TAMs increasing glucose availability for tumor cells [84]. Non-coding RNAs are crucial mediators of this interplay [85,86].

Cancer cells increase HIF-1α protein levels in response to hypoxia to counteract cellular stress. HIF-1α is well known for its ability to modify cancer cell metabolism by inducing the overexpression of different glycolytic enzymes [87]. In healthy cells, PHD2 rapidly hydroxylates and degrades HIF-1α through the ubiquitin/proteasome pathway [88]. However, in cancer cells, HIF-1α degradation is prevented, leading to cancer cell metabolic switch and tumor progression [89]. Chen et al. demonstrated that TAMs isolated from breast cancer tissue release extracellular vesicles (EVs) containing the lncRNA HISLA, which enters tumor cells and stabilizes HIF-1α leading to aerobic glycolysis upregulation. Mechanistically, HISLA functions by directly interacting with PHD2 and inhibiting its hydroxylase activity on HIF-1α, leading to its stabilization [77].

Lactate dehydrogenase B (LDHB) is a glycolysis-related enzyme that catalyzes the interconversion of pyruvate and lactate, whose function in macrophages has been poorly investigated [59]. Frank et al. demonstrated that miR-375 derived from breast cancer cells is captured by TAMs, where it reduces LDHB expression levels, likely by directly targeting its transcript. The decrease in LDHB leads to the upregulation of aerobic glycolysis and lactate production in TAMs. The downregulation of LDHB also results in the activation of SREBP2, an enzyme that regulates cholesterol synthesis. The increased production of lactate and sterol by TAMs in the TME boosts tumor progression by stimulating cancer cell proliferation and survival. Therefore, miR-375 can be a promising target to selectively interfere with TAM tumor-promoting functions by reverting their metabolic status [59].

MiR-503-3p is an miRNA upregulated in breast cancer patients with a poor prognosis [90]. Huang et al. reported that exosomal miR-503-3p secreted by TAMs directly targets DACT2 transcript, a tumor suppressor gene acting as an antagonist of β-catenin in BC cells. The inhibition of miR-503-3p upregulates DACT2 and inhibits Wnt/ β-catenin pathway in BC cells, resulting in glycolysis suppression and mitochondrial oxidative phosphorylation increase [60].

The inhibition of miR-33 regulates macrophage metabolism and M2-polarization by reducing glycolysis and promoting Fatty Acid Oxidation (FAO) in atherosclerosis [91]. Moradi-Chaleshtori et al. employed the delivery of 4T1 BC cell-derived exosomes containing miR-33 to macrophages to regulate their polarization. MiR-33 delivery resulted in macrophage polarization switch from M2 to M1 phenotype, and the upregulation of the M1 markers Nos2, Irf5, CD86, TNF-α, and IL-1β. The delivery of miR-33 induces M1 macrophage polarization also by regulating TAM metabolism. These M1-reprogrammed macrophages acquired the ability to inhibit 4T1 cell migration and invasion, identifying miR-33 delivery as a potential drug treatment for breast cancer [61].

Adipocyte/macrophage fatty acid-binding proteins (FABPs) are lipid chaperons that integrate inflammatory and metabolic responses in macrophages [92]. The upregulation of FABPs increases the pro-tumoral activity of a subtype of TAMs (CD11b+F4/80+Ly6C-MHCII-CD11c-) by upregulating the IL-6/STAT3 pathway via the modulation of NFkB/miR-29b. Mechanistically, FABPs downregulate miR-29b in TAMs via an increased NFkB activity, releasing the inhibitory effect of miR-29b on the pro-tumoral factor STAT3 and inducing macrophage M2 polarization. Therefore, the authors proposed FABPs as new prognostic markers and potential therapeutic targets in breast cancers [62].

**Figure 2:**
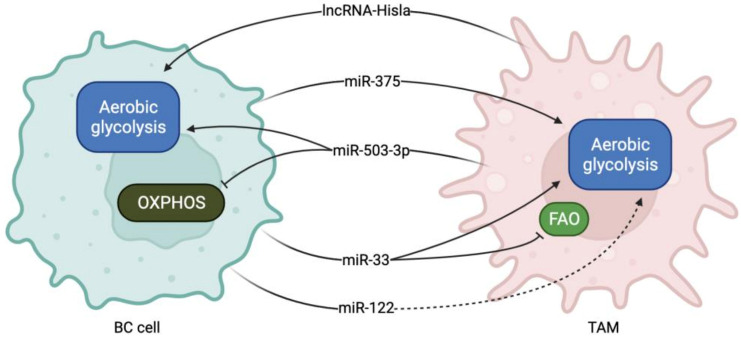
miRNAs and lncRNAs involved in TAM-BC crosstalk during metabolism reprogramming. LncRNA Hisla and miR-503-3p are secreted from TAMs and collected by BC cells, where they promote aerobic glycolysis and suppress OXPHOS. Instead, miR-375, miR-33 and miR-122 are secreted by BC cells and promote glycolysis upregulation and FAO inhibition in TAMs.

Breast cancer cells secrete miR-122, which suppresses glucose metabolism by reducing the glycolytic enzyme pyruvate kinase expression in the premetastatic niche. In this manner, miR-122 prepares the environment for the tumor cell arrival by increasing glucose availability and thus favoring cancer cell proliferation and dissemination. Indeed, the authors showed that miR-122 secreted from the primary tumor could affect glucose metabolism in the lungs and brain before metastases onset [63]. In this context, miR-122 may also influence macrophage metabolism and affect their pro-tumoral phenotype in the premetastatic niche. Figure 2, Table 1, and Table 2 summarize the role of miRNAs and lncRNAs in TAM and BC cell metabolic reprogramming during cancer progression.

## 4. Non-Coding RNAs in the Regulation of TAM-Induced Angiogenesis

The formation of new blood vessels is indispensable for the successful growth of tumor cells, which require a considerable supply of nutrients, oxygen, and other factors to sustain a high growth rate [93,94]. The increased blood vessel density in the tumor environment allows cancer cells to intravasate and enter circulation, the primary event for metastasis formation [93,94]. The direct correlation between TAM presence and a pro-angiogenic environment has been extensively documented in breast cancer [95,96]. In this context, cancer cells reprogram TAMs to a pro-angiogenic phenotype to support all the stages for blood vessel formation. Indeed, TAMs promote ECM remodeling through metalloproteinase secretion and stimulate endothelial cell proliferation and migration by secreting growth factors such as EGF, VEGF, and pro-angiogenic cytokines such as TNFα, CCL8, and IL-8 [95,97]. NcRNAs control TAM and BC cell angiogenic properties by regulating the expression of growth factors and the release of cytokines in the TME [97,98].

Our group recently demonstrated that ID4 expression in breast cancer cells drives TAM reprogramming by increasing the expression of two pro-angiogenic factors, granulin (GRN) and HIF. We demonstrated that ID4 expression in BC cells mediates the release of VEGF, which acts in a paracrine manner and promotes the downregulation of the anti-angiogenic miR-107, miR-15b, and miR-195 in macrophages. The downregulation of these miRNAs leads to GRN and HIF-1 expression in macrophages, promoting their pro-angiogenic abilities and breast cancer progression [64,99,100].

M2 macrophages also secrete factors that regulate BC cell pro-angiogenic activity. Dong et al. demonstrated that VEGF secreted by M2 macrophages induces the upregulation of the lncRNA PCAT6 in human BC cells. PCAT6 induces the expression of VEGFR2, leading to the activation of the Akt/mTOR pathway. Mechanistically, PCAT6 recruits the ubiquitin protease USP14 on VEGFR2 inhibiting its proteasome-mediated degradation. The activation of the Akt/mTOR pathway in BC cells triggers a pro-angiogenic response, both in vivo and in vitro, facilitating their tumorigenic behavior [78].

The administration of ncRNAs has emerged as a potential therapeutic strategy to stop tumor progression through angiogenesis suppression [101]. MiR-29b is a tumor suppressor miRNA in many cancers, where it regulates cell proliferation, apoptosis, and differentiation, and its expression also decreases in breast cancer tissue compared with normal mammary tissue [65]. Li et al. showed that the systemic administration of miR-29b in a mouse model of breast cancer significantly suppresses tumor growth by reducing angiogenesis. In BC cells, miR-29b downregulates Akt3 protein expression, which is known to induce VEGF and to promote angiogenesis. At the same time, the authors observed that the TAM infiltration was significantly reduced after miR-29b administration, indicating that miR-29b directly or indirectly acts on macrophage recruitment. The reduced number of TAMs further mitigates the secretion of pro-angiogenic factors in the TME [65].

MiR-155 is a well-known oncomiRNA induced by hypoxia and VEGF, which can promote TAM recruitment in breast cancer [102]. Kong et al. reported that the overexpression of miR-155 induces angiogenesis in a mouse model of breast cancer by directly targeting VHL mRNA. VHL targets the members of HIFα family; thus, its downregulation results in HIF1α and HIF2α expression and the induction of angiogenesis. In addition, miR-155 overexpression results in a massive TAM infiltration, although the mechanisms regulating this process have not been clarified yet [66]. TAMs can, in turn, produce miR-155 and miR-155-stimulating factors, such as VEGF, causing a positive feedback loop that boosts angiogenesis [103,104].

MiR-21 and miR-29a are two potent pro-angiogenic factors involved in TAM reprogramming. Mathsyaraja et al. showed that these factors are upregulated in a CSF1-ETS2-dependent pathway in infiltrating myeloid cells from a metastatic model of murine breast cancer. MiR-21 and miR-29a mediate the pro-angiogenic switch in TAMs by downregulating the expression of the anti-angiogenic genes Pdcd4, Spry1, Timp3 Col4a2, and Sparc. The expression of miR-21 and miR-29a also correlates with the upregulation of M2 markers in macrophages and with an increased TAM pro-tumoral potential [67]. Table 1 and Table 2 outline the roles of miRNAs and lncRNAs in TAM and BC cell pro-angiogenic activity during breast cancer progression.

## 5. Non-Coding RNAs in the Crosstalk between TAMs and BC Cells during Metastasis Formation

The metastatic spreading of cancer cells requires the cooperation between cancer cells and the TME [1]. Before metastasis onset, cancer cells acquire a motile mesenchymal phenotype by undergoing EMT, entering blood vessels, and finally extravasating and invading other tissues where they need to find a favorable niche to grow [105]. TAMs foster metastasis formation by promoting cancer cell EMT, angiogenesis and ECM remodeling, increasing the chances for cancer cells to extravasate and enter the circulation. Furthermore, TAMs provide factors to limit the immune response in the pre-metastatic niche to prepare the microenvironment for the arrival of metastatic cancer cells. [13,106]. Sanchez-Gonzalez et al. showed that miR-149 downregulation in BC cells promotes the secretion of CSF1, a cytokine that stimulates the secretion of EGFR ligands by TAMs. EGFR ligands then bind to the receptor expressed by cancer cells and activate the EGFR pathway, increasing BC cells’ malignant behavior and resulting in lung metastasis formation in an SCID mouse orthotopically injected with human BC cells. MiR-149 downregulation also favors tumor progression by increasing TAM recruitment to the tumor site [39]. MiR-100 expression in TAMs increases IL-1ra secretion via the modulation of the mTOR-Stat5a pathway. The authors showed that IL-1ra increases cancer cell invasive ability and stemness by inducing the activation of the Hedgehog signal pathway. In vivo experiments finally showed that miR-100 inhibition significantly reduced lung metastasis formation in a 4T1 BC mouse model [48]. Long noncoding RNA associated with breast cancer brain metastasis (lnc-BM) is upregulated in metastatic cells where it induces the expression of CCL2 via the activation of the JAK2/STAT3 pathway. The secretion of CCL2 recruits TAMs, which secrete oncostatin M (OSM) and IL-6, inducing an additional activation of the JAK2/STAT3 pathway, activating a positive feedback loop. The activation of the JAK/STAT pathway in cancer cells facilitates cell adhesion to capillaries and extravasation to the brain through the upregulation of the endothelial adhesive molecule ICAM. The treatment of a mouse xenograft model of BC with nanoparticles carrying lnc-BM siRNA inhibited brain metastasis formation [36].

Tumors are often characterized by a persistent and non-resolutive inflammation that correlates with a poor prognosis [107]. Guo et al. demonstrated that the delivery of miR-183-5p from 4T1 BC cells to macrophages mediated by exosomes increases the secretion of pro-inflammatory cytokines IL-1b, IL-6, and TNF-a from macrophages. Mechanistically, miR-183-5p targets PPP2CA in TAMs, leading to the upregulation of the NF-kB pathway, which is responsible for the activation of the pro-inflammatory pathway. The release of pro-inflammatory cytokines in the TME is detrimental and results in tumor progression and lung metastasis. The authors finally showed that the injection of BC cells with downregulated miR-183-5p in a mouse model results in a significant reduction in lung metastasis [68]. Another study showed that exosomal miR-183-5p transmission from BC cells to TAMs targets and inhibits the H3K27 histone demethylase KDM6B, inhibiting the expression of M1-related pro-inflammatory genes. The co-administration of BC cells and TAMs pre-treated with exosomes from cells overexpressing miR-183-5p promoted lung metastasis in vivo, highlighting the ability of this miRNA to induce TAM-mediated metastasis formation [69].

Progranulin (PGRN) is a secreted glycoprotein upregulated in many human cancers [108]. Recently, it has been shown that TAMs isolated from PGRN-/- mice harboring breast cancer release exosomes that can affect BC cell behavior by reducing EMT and invasive ability. These TAM-released exosomes contained high levels of miR-5100, which could be taken up by BC cells targeting CXCL12, a chemokine known to promote BC cell invasiveness [70]. In an in vivo model of PY8119 breast cancer, the absence of PGRN resulted in a reduced grade of TAM infiltration and reduced lung metastasis formation [109].

**Figure 3:**
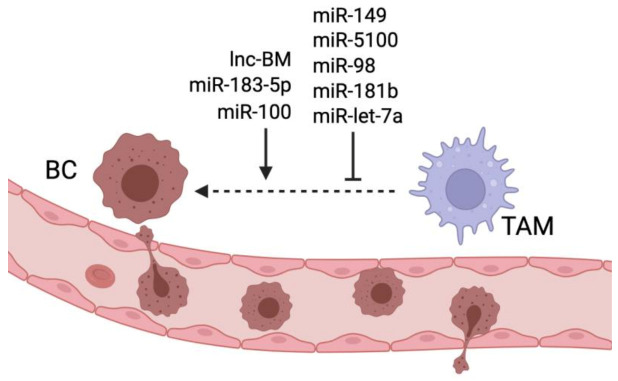
miRNAs and lncRNAs regulating TAM-induced breast cancer metastasis. Lnc-BM, miR-183-5p, and miR-100 promote TAM ability to stimulate breast cancer metastasis, while miR-149, miR-5100, miR-98, miR-181b and let-7a inhibit TAM- mediated metastasis formation.

TAMs are a primary source of CCL18, a chemokine associated with breast cancer progression and poor prognosis [110]. Lin et al. demonstrated that CCL18 decreases miR-98 expression in BC cells via Lin28b/ NF-κB activation, promoting the activation of the EMT pathway. To investigate the role of CCL18 on metastasis formation, the authors injected CCL18 in a mouse model harboring a breast tumor derived from MDA-MB-231 cells and observed an induction of lung and liver metastasis formation, together with the downregulation of miR-98 [71]. Another study showed that the ectopic overexpression of miR-181b in TAMs could counteract the pro-tumoral effects of TAM-released CCL18 by inactivating the NF-κB pathway in BC cells in vitro [72]. Let-7a is a downregulated miRNA in breast cancer that can inhibit the effects of CCL18 in BC cells by downregulating Lin28 expression. Therefore, let-7a delivery in BC tissue could be a potential strategy to limit the detrimental effects of TAMs [111]. Figure 3, Table 1, and Table 2 summarize the role of miRNAs and lncRNAs in TAM-induced breast cancer metastasis.

## 6. The Role of miRNAs and lncRNAs in TAM-Induced Chemoresistance in Breast Cancer

Drug resistance is the leading cause of relapse and death in cancer [112]. TAMs may play a significant role in response to chemotherapy by accumulating in the tumor tissue and mediating the pro-regenerative and anti-apoptotic responses that confer resistance to therapies to cancer cells [113]. In a recent study, Liu et al. reported that IRENA is the most upregulated lncRNA in TAMs after neoadjuvant chemotherapy in breast cancer patients. The authors demonstrated that chemotherapy activates the inflammatory response and upregulates the Jak1–STAT1-induced IRENA lncRNA. IRENA then induces the pro-inflammatory NF-κB pathway in TAMs by promoting the dimerization and phosphorylation of PKR, a protein that activates several inflammatory-related pathways [114]. Although inflammatory M1 macrophages are often associated with antitumor activity, the activation of the NF-κB pathway in the context of a chemotherapeutic treatment promoted TAM-mediated cancer cell chemoresistance. The authors showed that the IRENA knockdown in the PyMT murine model of breast cancer improved the cancer response to chemotherapy treatment [79].

MiR-770 is selectively overexpressed in chemo-sensitive breast cancer tissues and mediates doxorubicin sensitivity by regulating gene expression in BC cells and interfering with the TME. First, miR-770 promotes BC cells apoptosis after treatment with doxorubicin and reduces metastasis formation by directly targeting STMN1mRNA and inhibiting the EMT pathway. Secondly, BC cells can transfer miR-770 to TAMs, where it upregulates the M1 markers MCP-1, iNOS and CD80 while decreasing the expression of M2-macrophage markers. The M1-reprogrammed macrophages helped to reverse the BC cell’s resistance to doxorubicin and sensitize them to cell death, underlining the importance of the TME in the chemoresistance process [73].

The expression of the oncomiRNA miR-21 has been associated with trastuzumab and chemotherapy resistance in HER2-positive breast cancers [74]. MiR-21 mediates chemoresistance by acting in BC cells and TAM recruitment. In BC cells, miR-21 promotes survival by targeting PTEN and PDCD. In the TME, PTEN downregulation increased the secretion of IL-6 from BC cells, which correlated with higher TAM infiltration that contributed to the acquisition of chemoresistance in BC cells [74].

Tan et al. uncovered the role of miR-708 in chemo-resistant breast cancer stem cells. MiR-708 is downregulated in chemo-resistant stem cells, where it inhibits self-renewal by targeting the CD47. The overexpression of miR-708 in BC cells with increased chemosensitivity to docetaxel and increased macrophage phagocytic activity, restoring the immune-mediated elimination of chemo-resistant cells [75].

MiR-100 expression in TAMs increases IL-1ra secretion via the mTOR-Stat5a pathway. The release of IL-1ra in the TME facilitates cancer stem cell development via the activation of the Hedgehog pathway and promotes resistance to cisplatin treatment. The inhibition of miR-100 in a 4T1 breast cancer model significantly improved the cisplatin response, resulting in reduced metastasis formation [48].

Breast cancer cells resistant to adriamycin secrete exosomes containing miR-222, which are captured by macrophages, promoting M2 polarization by targeting PTEN and inducing the Akt pathway. By inducing M2 macrophages, chemo-resistant cells modify the TME to sustain tumor growth and metastasis formation [76].

The lncRNA LINC00337 is upregulated in breast cancer cells and promotes paclitaxel resistance by affecting the TAM phenotype. LINC00337 promotes CCL2, IL-13, and M-CSF secretion inducing macrophage recruitment and M2 polarization, as demonstrated by the upregulation of the M2 markers CD163 and ARGM2 macrophages, which then increase the malignant behavior of chemo-resistant cancer cells by promoting their survival and migratory abilities [80]. Table 1 and Table 2 recapitulate the role of ncRNAs in TAM-induced breast cancer chemoresistance.

## 7. Conclusions

Breast cancer is the most common tumor in women worldwide and a very heterogeneous disease [115]. Although the prognosis of non-metastatic breast cancer is generally favorable, the most aggressive forms remain incurable, and, in the case of chemo-resistant breast cancers, the treatments may alleviate the symptoms rather than eradicate the disease [115]. Therefore, new therapeutic approaches considering the molecular heterogeneities of the different breast cancer subtypes are needed to improve patients’ life expectancies [116]. The immunosuppressive and tumor-promoting tumor microenvironment allows cancer cells to escape the immune-mediated killing mechanisms and provides pro-survival signals, laying the foundations for metastasis formation and chemoresistance [1,117]. TAMs represent one of the primary cell populations in the TME that contribute to each stage of cancer progression [7,118]. Deregulated ncRNAs in BC cells promote TAM recruitment and polarization to the M2 phenotype and educate TAMs to promote cancer progression. At the same time, ncRNA alteration in TAMs affects BC cell behavior [98]. This crosstalk can be mediated by ncRNAs secreted in the TME and collected by nearby cells or by factors released in the TME that control ncRNA expression in other cells. Different studies showed that targeting TAMs is beneficial for breast cancer. Therapies under investigation involve blocking TAM recruitment, eliminating TAMs already present in the TME, or interfering with their polarization status [119].

TAM elimination is challenging, as the drugs used for this purpose may not be selective for macrophages. The manipulation of ncRNA expression could be a potential strategy to interfere with TAM recruitment or to reprogram M2-polarized macrophages [119]. NcRNAs are often released inside exosomes, so one possibility is the use of antibodies to target exosomes from cancer cells that can be recognized for the expression of particular proteins [120]. Another possibility involves the use of small molecules such as Antisense Oligonucleotides (ASOs) or small interfering RNA (siRNA) that inhibit or degrade ncRNA molecules [121]. On the contrary, the delivery of anti-oncogenic ncRNAs through exosomes or nanoparticles might restore altered molecular pathways in cancer [120,122]. Today, the use of RNA-based therapies for cancer treatment is still premature, due to the difficulties in drug delivery and the risk of off-target effects. However, in the future, the manipulation of ncRNA expression, alone or in combination with other treatments, could be a promising therapeutic approach to interfere with TAM pro-tumoral functions in breast cancer and other cancers.

## Figures and Tables

**Table 1:** miRNAs involved in BC cells-TAM crosstalk during BC progression.

microRNA	Expression	Regulated Pathways	Biological Function	Reference
miR-375	Transferred from apoptotic BC cells to TAMs	Induces CCL2 secretion in BC cells and TNS3 and PXN downregulation in TAMs	Promotes TAM migration and recruitment	[34]
miR-149	Brain metastatic BC cells	Directly targets and inhibits CSF1	Limits TAM recruitment and lung metastasis; promotes M1 polarization	[39]
miR-200c	BC cells	Induces GM-CSF secretion	Induces M1 polarization	[41]
miR-200c	BC cells	Induces nPAI-2-mediated IL-10 secretion	Induces M2 polarization	[45]
miR-19a-3p	TAMs	Inhibits Fra1/ STAT3 pathway	Inhibits M2 polarization	[47]
miR-100	TAMs	Inhibits mTOR and induces STAT5-mediated IL1-ra secretion	Induces M2 polarization, lung metastasis and chemoresistance	[48]
miR-181a	TAMs	Inhibits C/EBPa and KLF6 expression	Induces M2 polarization	[54]
miR-720	TAMs	Directly targets and inhibits GATA3	Inhibits M2 polarization	[56]
Let-7b	Ectopically administered	Acts as an agonist of TLR7 in TAMs and inhibits IL-10 secretion	Inhibits M2 polarization	[58]
miR-375	Transferred from BC cells to TAMs	Inhibits LDHB expression Induces SREBP2 activation	Induces aerobic glycolysis and lactate production Induces cholesterol synthesis	[59]
miR-503-3p	Transferred from TAMs cells to BC cells	Activates Wnt/β-cathenin pathway by directly targeting DACT2	Induces glycolysis and inhibits OXPHOS	[60]
miR-33	Ectopically administered	Stimulates M1-related cytokine production	Induces M1 polarization. induces glycolysis and inhibits FAO	[61]
miR-29b	TAMs	Inhibits STAT3 pathway	Inhibits M2 polarization	[62]
miR-122	BC cells	PK downregulation	Increases glucose availability in the pre-metastatic niche for cancer cells	[63]
miR-107 miR-15b	TAMs	Inhibits HIF1 and GRN expression	Inhibits TAM pro-angiogenic functions	[64]
miR-29b	BC cells	Inhibits VEGF secretion by downregulating Akt3	Inhibits angiogenesis	[65]
miR-155	BC cells/ TAMs	Directly targets and inhibits VHL	Induces angiogenesis	[66]
miR-21 miR-29a	TAMs	Inhibit Pdcd4, Spry1, Timp3, Col4a2, and Sparc expression	Induces TAM pro-angiogenic reprogamming	[67]
miR-183-5p	Transferred from BC cells to TAMs	Directly targets and inhibits PPP2CA inducing NFkB activation	Promotes the formation of a pro-inflammatory TME and induces lung metastasis	[68]
miR-183-5p	Transferred from BC cells to TAMs	Inhibits KDM6B histone demethylase	Induces M2 polarization and promotes lung metastasis	[69]
miR-5100	Transferred from TAMs to BC cells in PGRN-/- mice	Inhibits CXCL12 production	Reduces TAM infiltration; reduces EMT and lung metastasis	[70]
miR-98	BC cells	Inhibits EMT	Inhibits lung and liver metastasis	[71]
miR-181b	Ectopically administred	Downregulates NFkB pathway	Inhibits lung metastasis	[72]
miR-770	BC cells Transferred from BC cells to TAMs	Inhibits STMN1 expression Induces M1 genes	Inhibits EMT and promotes cell death Promotes M1 polarization	[73]
miR-21	BC cells	Inhibits PTEN and PDCD4 expression	Induces chemoresistance and TAM infiltration	[74]
miR-708	Chemo-resistant BC stem cells	Directly targets and inhibits CD47	Inhibits cancer stem cell self-renewal and increases M1 phagocitic activity reducing chemoresistance	[75]
miR-222	Transferred from BC cells to TAMs	Induces Akt pathway via PTEN downregulation	Induces M2 polarization and cancer chemoresistance	[76]

**Table 2:** lncRNAs involved in BC cells-TAM crosstalk during BC progression.

lncRNA	Expression	Regulated Pathways	Biological Function	References
lnc-BM	Brain metastatic BC cells	Upregulates JAK2/STAT3 pathway	Promotes TAM recruitment and brain metastasis formation	[36]
linc-00514	BC cells	Induces STAT3-mediated Jagge1 upregulation	Promotes M2 polarization	[49]
lnc-Xist	TAMs	Sponges miR-101 and induces C/EBPα and KLF6 expression	Promotes M1 polarization	[51]
lnc-GNAS-AS1	BC cells/ TAMs	Sponges miR-433-3p and induces GATA3 expression	Promotes M2 polarization	[55]
lnc-Hisla	Transferred from TAMs to BC cells	Upregulates HIF1α expression by inhibiting PHD2	Induces aerobic glycolysis	[77]
lnc-PCAT6	BC cells	Inhibits VEGFR2 degradation	Induces angiogenesis	[78]
lnc-IRENA	TAMs	Induces PKR-mediated NFkB pathway activation	Induces BC chemoresistance	[79]
linc-00337	BC cells	Promotes CCL12, IL-13, M-CSF secretion	Induces chemoresistance, and M2 polarization	[80]

## References

[1] Quail D.F., Joyce J.A. (2013). Microenvironmental regulation of tumor progression and metastasis. Nat. Med..

[2] Whiteside T.L. (2008). The tumor microenvironment and its role in promoting tumor growth. Oncogene.

[3] Wang M., Zhao J., Zhang L., Wei F., Lian Y., Wu Y., Gong Z., Zhang S., Zhou J., Cao K. (2017). Role of tumor microenvironment in tumorigenesis. J. Cancer.

[4] Brassart-Pasco S., Brézillon S., Brassart B., Ramont L., Oudart J.-B., Monboisse J.C. (2020). Tumor Microenvironment: Extracellular Matrix Alterations Influence Tumor Progression. Front. Oncol..

[5] Wang J., Li D., Cang H., Guo B. (2019). Crosstalk between cancer and immune cells: Role of tumor-associated macrophages in the tumor microenvironment. Cancer Med..

[6] Mantovani A., Marchesi F., Malesci A., Laghi L., Allavena P. (2017). Tumour-associated macrophages as treatment targets in oncology. Nat. Rev. Clin. Oncol..

[7] Pan Y., Yu Y., Wang X., Zhang T. (2020). Tumor-Associated Macrophages in Tumor Immunity. Front. Immunol..

[8] Shapouri-Moghaddam A., Mohammadian S., Vazini H., Taghadosi M., Esmaeili S.-A., Mardani F., Seifi B., Mohammadi A., Afshari J.T., Sahebkar A. (2018). Macrophage plasticity, polarization, and function in health and disease. J. Cell. Physiol..

[9] Orecchioni M., Ghosheh Y., Pramod A.B., Ley K. (2019). Macrophage Polarization: Different Gene Signatures in M1(LPS+) vs. Classically and M2(LPS–) vs. Alternatively Activated Macrophages. Front. Immunol..

[10] Jayasingam S.D., Citartan M., Thang T.H., Mat Zin A.A., Ang K.C., Ch’ng E.S. (2020). Evaluating the Polarization of Tumor-Associated Macrophages Into M1 and M2 Phenotypes in Human Cancer Tissue: Technicalities and Challenges in Routine Clinical Practice. Front. Oncol..

[11] Wynn T.A., Vannella K.M. (2016). Macrophages in Tissue Repair, Regeneration, and Fibrosis. Immunity.

[12] Chen Y., Tan W., Wang C. (2018). Tumor-associated macrophage-derived cytokines enhance cancer stem-like characteristics through epithelial–mesenchymal transition. OncoTargets Ther..

[13] Lin Y., Xu J., Lan H. (2019). Tumor-associated macrophages in tumor metastasis: Biological roles and clinical therapeutic applications. J. Hematol. Oncol..

[14] Oshi M., Tokumaru Y., Asaoka M., Yan L., Satyananda V., Matsuyama R., Matsuhashi N., Futamura M., Ishikawa T., Yoshida K. (2020). M1 Macrophage and M1/M2 ratio defined by transcriptomic signatures resemble only part of their conventional clinical characteristics in breast cancer. Sci. Rep..

[15] Anastasiadou E., Jacob L.S., Slack F.J. (2018). Non-coding RNA networks in cancer. Nat. Rev. Cancer.

[16] Grillone K., Riillo C., Scionti F., Rocca R., Tradigo G., Guzzi P.H., Alcaro S., Di Martino M.T., Tagliaferri P., Tassone P. (2020). Non-coding RNAs in cancer: Platforms and strategies for investigating the genomic “dark matter”. J. Exp. Clin. Cancer Res..

[17] O’Brien J., Hayder H., Zayed Y., Peng C. (2018). Overview of MicroRNA Biogenesis, Mechanisms of Actions, and Circulation. Front. Endocrinol..

[18] Ha M., Kim V.N. (2014). Regulation of microRNA biogenesis. Nat. Rev. Mol. Cell Biol..

[19] Müller M., Fazi F., Ciaudo C. (2020). Argonaute Proteins: From Structure to Function in Development and Pathological Cell Fate Determination. Front. Cell Dev. Biol..

[20] Bellissimo T., Tito C., Ganci F., Sacconi A., Masciarelli S., Di Martino G., Porta N., Cirenza M., Sorci M., De Angelis L. (2019). Argonaute 2 drives miR-145-5p-dependent gene expression program in breast cancer cells. Cell Death Dis..

[21] Dahariya S., Paddibhatla I., Kumar S., Raghuwanshi S., Pallepati A., Gutti R.K. (2019). Long non-coding RNA: Classification, biogenesis and functions in blood cells. Mol. Immunol..

[22] Iaiza A., Tito C., Ianniello Z., Ganci F., Laquintana V., Gallo E., Sacconi A., Masciarelli S., De Angelis L., Aversa S. (2021). METTL3-dependent MALAT1 delocalization drives c-Myc induction in thymic epithelial tumors. Clin. Epigenetics.

[23] Tito C., Ganci F., Sacconi A., Masciarelli S., Fontemaggi G., Pulito C., Gallo E., Laquintana V., Iaiza A., De Angelis L. (2020). LINC00174 is a novel prognostic factor in thymic epithelial tumors involved in cell migration and lipid metabolism. Cell Death Dis..

[24] Klinge C.M. (2018). Non-Coding RNAs in Breast Cancer: Intracellular and Intercellular Communication. Non-Coding RNA.

[25] Howard E.W., Yang X. (2018). microRNA Regulation in Estrogen Receptor-Positive Breast Cancer and Endocrine Therapy. Biol. Proced. Online.

[26] Sharma S., Patnaik P.K., Aronov S., Kulshreshtha R. (2016). ApoptomiRs of Breast Cancer: Basics to Clinics. Front. Genet..

[27] Zare M., Bastami M., Solali S., Alivand M.R. (2017). Aberrant miRNA promoter methylation and EMT-involving miRNAs in breast cancer metastasis: Diagnosis and therapeutic implications. J. Cell. Physiol..

[28] Shimono Y., Mukohyama J., Nakamura S.-I., Minami H. (2015). MicroRNA Regulation of Human Breast Cancer Stem Cells. J. Clin. Med..

[29] Amelio I., Bernassola F., Candi E. (2020). Emerging roles of long non-coding RNAs in breast cancer biology and management. Semin. Cancer Biol..

[30] Chen Y.G., Satpathy A.T., Chang H.Y. (2017). Gene regulation in the immune system by long noncoding RNAs. Nat. Immunol..

[31] Curtale G., Rubino M., Locati M. (2019). MicroRNAs as Molecular Switches in Macrophage Activation. Front. Immunol..

[32] Hao Q., Vadgama J.V., Wang P. (2020). CCL2/CCR2 signaling in cancer pathogenesis. Cell Commun. Signal..

[33] Lim S.Y., Yuzhalin A., Gordon-Weeks A.N., Muschel R.J. (2016). Targeting the CCL2-CCR2 signaling axis in cancer metastasis. Oncotarget.

[34] Frank A.-C., Ebersberger S., Fink A.F., Lampe S., Weigert A., Schmid T., Ebersberger I., Syed S.N., Brüne B. (2019). Apoptotic tumor cell-derived microRNA-375 uses CD36 to alter the tumor-associated macrophage phenotype. Nat. Commun..

[35] Kitamura T., Qian B.-Z., Soong D., Cassetta L., Noy R., Sugano G., Kato Y., Li J., Pollard J.W. (2015). CCL2-induced chemokine cascade promotes breast cancer metastasis by enhancing retention of metastasis-associated macrophages. J. Exp. Med..

[36] Wang S., Liang K., Hu Q., Li P., Qingsong H., Yang Y., Yao J., Mangala L.S., Li C., Yang W. (2017). JAK2-binding long noncoding RNA promotes breast cancer brain metastasis. J. Clin. Investig..

[37] Cannarile M.A., Weisser M., Jacob W., Jegg A.-M., Ries C.H., Rüttinger D. (2017). Colony-stimulating factor 1 receptor (CSF1R) inhibitors in cancer therapy. J. Immunother. Cancer.

[38] Elin R., Rebecca Dale U., Stein Harald J., Lill-Tove B. (2015). Macrophage-colony stimulating factor (CSF1) predicts breast cancer progression and mortality. Anticancer Res..

[39] Sánchez-González I., Bobien A., Molnar C., Schmid S., Strotbek M., Boerries M., Busch H., Olayioye M.A. (2020). miR-149 Suppresses Breast Cancer Metastasis by Blocking Paracrine Interactions with Macrophages. Cancer Res..

[40] Chen Y., Zhang L. (2017). Members of the microRNA-200 family are promising therapeutic targets in cancer. Exp. Ther. Med..

[41] Williams M.M., Christenson J.L., O’Neill K.I., Hafeez S.A., Ihle C.L., Spoelstra N.S., Slansky J.E., Richer J.K. (2021). MicroRNA-200c restoration reveals a cytokine profile to enhance M1 macrophage polarization in breast cancer. NPJ Breast Cancer.

[42] Hamilton J.A. (2019). GM-CSF-Dependent Inflammatory Pathways. Front. Immunol..

[43] Le T.N.M., Hamar P., Guo C., Basar E., Perdigão-Henriques R., Balaj L., Lieberman J. (2014). miR-200–containing extracellular vesicles promote breast cancer cell metastasis. J. Clin. Investig..

[44] Tuomarila M., Luostari K., Soini Y., Kataja V., Kosma V.-M., Mannermaa A. (2014). Overexpression of MicroRNA-200c Predicts Poor Outcome in Patients with PR-Negative Breast Cancer. PLoS ONE.

[45] Meng Z., Zhang R., Wang Y., Zhu G., Jin T., Li C., Zhang S. (2019). miR-200c/PAI-2 promotes the progression of triple negative breast cancer via M1/M2 polarization induction of macrophage. Int. Immunopharmacol..

[46] Masjedi A., Hashemi V., Hojjat-Farsangi M., Ghalamfarsa G., Azizi G., Yousefi M., Jadidi-Niaragh F. (2018). The significant role of interleukin-6 and its signaling pathway in the immunopathogenesis and treatment of breast cancer. Biomed. Pharmacother..

[47] Yang J., Zhang Z., Chen C., Liu Y., Si Q., Chuang T.-H., Li N., Gomezcabrero A., Reisfeld R.A., Xiang R. (2013). MicroRNA-19a-3p inhibits breast cancer progression and metastasis by inducing macrophage polarization through downregulated expression of Fra-1 proto-oncogene. Oncogene.

[48] Wang W., Liu Y., Guo J., He H., Mi X., Chen C., Xie J., Wang S., Wu P., Cao F. (2018). miR-100 maintains phenotype of tumor-associated macrophages by targeting mTOR to promote tumor metastasis via Stat5a/IL-1ra pathway in mouse breast cancer. Oncogenesis.

[49] Tao S., Chen Q., Lin C., Dong H. (2020). Linc00514 promotes breast cancer metastasis and M2 polarization of tumor-associated macrophages via Jagged1-mediated notch signaling pathway. J. Exp. Clin. Cancer Res..

[50] Zheng R., Lin S., Guan L., Yuan H., Liu K., Liu C., Ye W., Liao Y., Jia J., Zhang R. (2018). Long non-coding RNA XIST inhibited breast cancer cell growth, migration, and invasion via miR-155/CDX1 axis. Biochem. Biophys. Res. Commun..

[51] Zhao Y., Yu Z., Ma R., Zhang Y., Zhao L., Yan Y., Lv X., Zhang L., Su P., Bi J. (2021). lncRNA-Xist/miR-101-3p/KLF6/C/EBPα axis promotes TAM polarization to regulate cancer cell proliferation and migration. Mol. Ther.-Nucleic Acids.

[52] Yang C., Tabatabaei S.N., Ruan X., Hardy P. (2017). The Dual Regulatory Role of MiR-181a in Breast Cancer. Cell. Physiol. Biochem..

[53] Jiang M., Zhang W., Zhang R., Liu P., Ye Y., Yu W., Guo X., Yu J. (2020). Cancer exosome-derived miR-9 and miR-181a promote the development of early-stage MDSCs via interfering with SOCS3 and PIAS3 respectively in breast cancer. Oncogene.

[54] Bi J., Zeng X., Zhao L., Wei Q., Yu L., Wang X., Yu Z., Cao Y., Shan F., Wei M. (2016). miR-181a Induces Macrophage Polarized to M2 Phenotype and Promotes M2 Macrophage-mediated Tumor Cell Metastasis by Targeting KLF6 and C/EBPα. Mol. Ther.-Nucleic Acids.

[55] Liu S.-Q., Zhou Z.-Y., Dong X., Guo L., Zhang K.-J. (2020). LncRNA GNAS-AS1 facilitates ER+ breast cancer cells progression by promoting M2 macrophage polarization via regulating miR-433-3p/GATA3 axis. Biosci. Rep..

[56] Zhong Y., Yi C. (2016). MicroRNA-720 suppresses M2 macrophage polarization by targeting GATA. Biosci. Rep..

[57] Mohapatra S., Pioppini C., Ozpolat B., Calin G.A. (2021). Non-coding RNAs regulation of macrophage polarization in cancer. Mol. Cancer.

[58] Huang Z., Gan J., Long Z., Guo G., Shi X., Wang C., Zang Y., Ding Z., Chen J., Zhang J. (2016). Targeted delivery of let-7b to reprogramme tumor-associated macrophages and tumor infiltrating dendritic cells for tumor rejection. Biomaterials.

[59] Frank A.-C., Raue R., Fuhrmann D.C., Sirait-Fischer E., Reuse C., Weigert A., Lütjohann D., Hiller K., Syed S.N., Brüne B. (2021). Lactate dehydrogenase B regulates macrophage metabolism in the tumor microenvironment. Theranostics.

[60] Huang S., Fan P., Zhang C., Xie J., Gu X., Lei S., Chen Z., Huang Z. (2021). Exosomal microRNA-503-3p derived from macrophages represses glycolysis and promotes mitochondrial oxidative phosphorylation in breast cancer cells by elevating DACT. Cell Death Discov..

[61] Moradi-Chaleshtori M., Bandehpour M., Heidari N., Mohammadi-Yeganeh S., Hashemi S.M. (2020). Exosome-mediated miR-33 transfer induces M1 polarization in mouse macrophages and exerts antitumor effect in 4T1 breast cancer cell line. Int. Immunopharmacol..

[62] Hao J., Yan F., Zhang Y., Triplett A., Zhang Y., Schultz D.A., Sun Y., Zeng J., Silverstein K.A., Zheng Q. (2018). Expression of Adipocyte/Macrophage Fatty Acid–Binding Protein in Tumor-Associated Macrophages Promotes Breast Cancer Progression. Cancer Res..

[63] Fong M.Y., Zhou W., Liu L., Alontaga A.Y., Chandra M., Ashby J., Chow A., O’Connor S.T.F., Li S., Chin A.R. (2015). Breast-cancer-secreted miR-122 reprograms glucose metabolism in premetastatic niche to promote metastasis. Nat. Cell Biol..

[64] Donzelli S., Milano E., Pruszko M., Sacconi A., Masciarelli S., Iosue I., Melucci E., Gallo E., Terrenato I., Mottolese M. (2018). Expression of ID4 protein in breast cancer cells induces reprogramming of tumour-associated macrophages. Breast Cancer Res..

[65] Li Y., Cai B., Shen L., Dong Y., Lu Q., Sun S., Liu S., Ma S., Ma P.X., Chen J. (2017). MiRNA-29b suppresses tumor growth through simultaneously inhibiting angiogenesis and tumorigenesis by targeting Akt. Cancer Lett..

[66] Kong W., He L., Richards E.J., Challa S., Xu C.-X., Permuthwey J., Lancaster J.M., Coppola D.M., Sellers T.A., Djeu J.Y. (2013). Upregulation of miRNA-155 promotes tumour angiogenesis by targeting VHL and is associated with poor prognosis and triple-negative breast cancer. Oncogene.

[67] Mathsyaraja H., Thies K., Taffany D.A., Deighan C., Liu T., Yu L., Fernandez S.A., Shapiro C.L., Otero J.P., Timmers C. (2014). CSF1-ETS2-induced microRNA in myeloid cells promote metastatic tumor growth. Oncogene.

[68] Guo J., Duan Z., Zhang C., Wang W., He H., Liu Y., Wu P., Wang S., Song M., Chen H. (2020). Mouse 4T1 Breast Cancer Cell–Derived Exosomes Induce Proinflammatory Cytokine Production in Macrophages via miR-183. J. Immunol..

[69] Xun J., Du L., Gao R., Shen L., Wang D., Kang L., Chen C., Zhang Z., Zhang Y., Yue S. (2021). Cancer-derived exosomal miR-138-5p modulates polarization of tumor-associated macrophages through inhibition of KDM6B. Theranostics.

[70] Yue S., Ye X., Zhou T., Gan D., Qian H., Fang W., Yao M., Zhang D., Shi H., Chen T. (2020). PGRN−/− TAMs-derived exosomes inhibit breast cancer cell invasion and migration and its mechanism exploration. Life Sci..

[71] Lin X., Chen L., Yao Y., Zhao R., Cui X., Chen J., Hou K., Zhang M., Su F., Chen J. (2015). CCL18-mediated down-regulation of miR98 and miR27b promotes breast cancer metastasis. Oncotarget.

[72] Wang L., Wang Y.-X., Chen L.-P., Ji M.-L. (2016). Upregulation of microRNA-181b inhibits CCL18-induced breast cancer cell metastasis and invasion via the NF-κB signaling pathway. Oncol. Lett..

[73] Li Y., Liang Y., Sang Y., Song X., Zhang H., Liu Y., Jiang L., Yang Q. (2018). MiR-770 suppresses the chemo-resistance and metastasis of triple negative breast cancer via direct targeting of STMN. Cell Death Dis..

[74] De Mattos-Arruda L., Bottai G., Nuciforo P., Di Tommaso L., Giovannetti E., Peg V., Losurdo A., Pérez-Garcia J., Masci G., Corsi F. (2015). MicroRNA-21 links epithelial-to-mesenchymal transition and inflammatory signals to confer resistance to neoadjuvant trastuzumab and chemotherapy in HER2-positive breast cancer patients. Oncotarget.

[75] Tan W., Tang H., Jiang X., Ye F., Huang L., Shi D., Li L., Huang X., Li L., Xie X. (2019). Metformin mediates induction of miR-708 to inhibit self-renewal and chemoresistance of breast cancer stem cells through targeting CD47. J. Cell. Mol. Med..

[76] Chen W.-X., Wang D.-D., Zhu B., Zhu Y.-Z., Zheng L., Feng Z.-Q., Qin X.-H. (2021). Exosomal miR-222 from adriamycin-resistant MCF-7 breast cancer cells promote macrophages M2 polarization via PTEN/Akt to induce tumor progression. Aging.

[77] Chen F., Chen J., Yang L., Liu J., Zhang X., Zhang Y., Tu Q., Yin D., Lin D., Wong P.P. (2019). Extracellular vesicle-packaged HIF-1α-stabilizing lncRNA from tumour-associated macrophages regulates aerobic glycolysis of breast cancer cells. Nat. Cell Biol..

[78] Dong F., Ruan S., Wang J., Xia Y., Le K., Xiao X., Hu T., Wang Q. (2020). M2 macrophage-induced lncRNA PCAT6 facilitates tumorigenesis and angiogenesis of triple-negative breast cancer through modulation of VEGFR. Cell Death Dis..

[79] Liu J., Lao L., Chen J., Li J., Zeng W., Zhu X., Li J., Chen X., Yang L., Xing Y. (2021). The IRENA lncRNA converts chemotherapy-polarized tumor-suppressing macrophages to tumor-promoting phenotypes in breast cancer. Nat. Cance.

[80] Xing Z., Zhang M., Liu J., Liu G., Feng K., Wang X. (2021). LINC00337 induces tumor development and chemoresistance to paclitaxel of breast cancer by recruiting M2 tumor-associated macrophages. Mol. Immunol..

[81] Pavlova N.N., Thompson C.B. (2016). The Emerging Hallmarks of Cancer Metabolism. Cell Metab..

[82] Hsu P.P., Sabatini D.M. (2008). Cancer Cell Metabolism: Warburg and Beyond. Cell.

[83] Mehla K., Singh P.K. (2019). Metabolic Regulation of Macrophage Polarization in Cancer. Trends Cancer.

[84] Vitale I., Manic G., Coussens L.M., Kroemer G., Galluzzi L. (2019). Macrophages and Metabolism in the Tumor Microenvironment. Cell Metab..

[85] Li J., Lu Z., Zhang Y., Xia L., Su Z. (2021). Emerging roles of non-coding RNAs in the metabolic reprogramming of tumor-associated macrophages. Immunol. Lett..

[86] Zhang Y., Mao Q., Xia Q., Cheng J., Huang Z., Li Y., Chen P., Yang J., Fan X., Liang Y. (2021). Noncoding RNAs link metabolic reprogramming to immune microenvironment in cancers. J. Hematol. Oncol..

[87] Jun J.C., Rathore A., Younas H., Gilkes D., Polotsky V.Y. (2017). Hypoxia-Inducible Factors and Cancer. Curr. Sleep Med. Rep..

[88] Wielockx B., Meneses A.M. (2016). PHD2: From hypoxia regulation to disease progression. Hypoxia.

[89] Soni S., Padwad Y.S. (2017). HIF-1 in cancer therapy: Two decade long story of a transcription factor. Acta Oncol..

[90] Zhao Z., Fan X., Jiang L., Xu Z., Xue L., Zhan Q., Song Y. (2017). miR-503-3p promotes epithelial–mesenchymal transition in breast cancer by directly targeting SMAD2 and E-cadherin. J. Genet. Genom..

[91] Ouimet M., Ediriweera H.N., Gundra U.M., Sheedy F., Ramkhelawon B., Hutchison S.B., Rinehold K., Van Solingen C., Fullerton M.D., Cecchini K. (2015). MicroRNA-33–dependent regulation of macrophage metabolism directs immune cell polarization in atherosclerosis. J. Clin. Investig..

[92] Maeda K., Cao H., Kono K., Gorgun C.Z., Furuhashi M., Uysal K.T., Cao Q., Atsumi G., Malone H., Krishnan B. (2005). Adipocyte/macrophage fatty acid binding proteins control integrated metabolic responses in obesity and diabetes. Cell Metab..

[93] Lugano R., Ramachandran M., Dimberg A. (2020). Tumor angiogenesis: Causes, consequences, challenges and opportunities. Cell. Mol. Life Sci..

[94] Zuazo-Gaztelu I., Casanovas O. (2018). Unraveling the Role of Angiogenesis in Cancer Ecosystems. Front. Oncol..

[95] Riabov V., Gudima A., Wang N., Mickley A., Orekhov A., Kzhyshkowska J. (2014). Role of tumor associated macrophages in tumor angiogenesis and lymphangiogenesis. Front. Physiol..

[96] Lin E.Y., Pollard J.W. (2007). Tumor-Associated Macrophages Press the Angiogenic Switch in Breast Cancer: Figure 1. Cancer Res..

[97] Munir M., Kay M., Kang M., Rahman M., Al-Harrasi A., Choudhury M., Moustaid-Moussa N., Hussain F., Rahman S. (2021). Tumor-Associated Macrophages as Multifaceted Regulators of Breast Tumor Growth. Int. J. Mol. Sci..

[98] Han D., Fang Y., Guo Y., Hong W., Tu J., Wei W. (2019). The emerging role of long non-coding RNAs in tumor-associated macrophages. J. Cancer.

[99] Donzelli S., Sacconi A., Turco C., Gallo E., Milano E., Iosue I., Blandino G., Fazi F., Fontemaggi G. (2020). Paracrine Signaling from Breast Cancer Cells Causes Activation of ID4 Expression in Tumor-Associated Macrophages. Cells.

[100] Turco C., Donzelli S., Fontemaggi G. (2020). miR-15/107 microRNA Gene Group: Characteristics and Functional Implications in Cancer. Front. Cell Dev. Biol..

[101] Song X., Guo Y., Song P., Duan D., Guo W. (2021). Non-coding RNAs in Regulating Tumor Angiogenesis. Front. Cell Dev. Biol..

[102] Mattiske S., Suetani R.J., Neilsen P., Callen D. (2012). The Oncogenic Role of miR-155 in Breast Cancer. Cancer Epidemiology Biomarkers Prev..

[103] Li X., Chen Z., Ni Y., Bian C., Huang J., Chen L., Xie X., Wang J. (2021). Tumor-associated macrophages secret exosomal miR-155 and miR-196a-5p to promote metastasis of non-small-cell lung cancer. Transl. Lung Cancer Res..

[104] Bruning U., Cerone L., Neufeld Z., Fitzpatrick S.F., Cheong A., Scholz C.C., Simpson D.A., Leonard M.O., Tambuwala M.M., Cummins E.P. (2011). MicroRNA-155 Promotes Resolution of Hypoxia-Inducible Factor 1 Activity during Prolonged Hypoxia. Mol. Cell. Biol..

[105] Fares J., Fares M.Y., Khachfe H.H., Salhab H.A., Fares Y. (2020). Molecular principles of metastasis: A hallmark of cancer revisited. Signal Transduct. Target. Ther..

[106] Condeelis J., Pollard J.W. (2006). Macrophages: Obligate Partners for Tumor Cell Migration, Invasion, and Metastasis. Cell.

[107] Zhao H., Wu L., Yan G., Chen Y., Zhou M., Wu Y., Li Y. (2021). Inflammation and tumor progression: Signaling pathways and targeted intervention. Signal Transduct. Target. Ther..

[108] Arechavaleta-Velasco F., Perez-Juarez C.E., Gerton G.L., Diaz-Cueto L. (2017). Progranulin and its biological effects in cancer. Med. Oncol..

[109] Ahirwar D.K., Nasser M.W., Ouseph M.M., Elbaz M., Cuitiño M.C., Kladney R.D., Varikuti S., Kaul K., Satoskar A.R., Ramaswamy B. (2018). Fibroblast-derived CXCL12 promotes breast cancer metastasis by facilitating tumor cell intravasation. Oncogene.

[110] Chen J., Yao Y., Gong C., Yu F., Su S., Chen J., Liu B., Deng H., Wang F., Lin L. (2011). CCL18 from Tumor-Associated Macrophages Promotes Breast Cancer Metastasis via PITPNM. Cancer Cell.

[111] Wang L., Wang Y.-X., Zhang D.-Z., Fang X.-J., Sun P.-S., Xue H.-C. (2016). Let-7a mimic attenuates CCL18 induced breast cancer cell metastasis through Lin 28 pathway. Biomed. Pharmacother..

[112] Vasan N., Baselga J., Hyman D.M. (2019). A view on drug resistance in cancer. Nature.

[113] Larionova I., Cherdyntseva N., Liu T., Patysheva M., Rakina M., Kzhyshkowska J. (2019). Interaction of tumor-associated macrophages and cancer chemotherapy. OncoImmunology.

[114] Kang R., Tang D. (2012). PKR-Dependent Inflammatory Signals. Sci. Signal..

[115] Waks A.G., Winer E.P. (2019). Breast Cancer Treatment: A Review. JAMA.

[116] Padh H. (2004). Novel therapeutics. Curr. Sci..

[117] Baghban R., Roshangar L., Jahanban-Esfahlan R., Seidi K., Ebrahimi-Kalan A., Jaymand M., Kolahian S., Javaheri T., Zare P. (2020). Tumor microenvironment complexity and therapeutic implications at a glance. Cell Commun. Signal..

[118] Cassetta L., Fragkogianni S., Sims A.H., Swierczak A., Forrester L.M., Zhang H., Soong D.Y.H., Cotechini T., Anur P., Lin E.Y. (2019). Human Tumor-Associated Macrophage and Monocyte Transcriptional Landscapes Reveal Cancer-Specific Reprogramming, Biomarkers, and Therapeutic Targets. Cancer Cell.

[119] Xiang X., Wang J., Lu D., Xu X. (2021). Targeting tumor-associated macrophages to synergize tumor immunotherapy. Signal Transduct. Target. Ther..

[120] Li C., Ni Y.-Q., Xu H., Xiang Q.-Y., Zhao Y., Zhan J.-K., He J.-Y., Li S., Liu Y.-S. (2021). Roles and mechanisms of exosomal non-coding RNAs in human health and diseases. Signal Transduct. Target. Ther..

[121] Winkle M., El-Daly S.M., Fabbri M., Calin G.A. (2021). Noncoding RNA therapeutics — challenges and potential solutions. Nat. Rev. Drug Discov..

[122] Ingenito F., Roscigno G., Affinito A., Nuzzo S., Scognamiglio I., Quintavalle C., Condorelli G. (2019). The Role of Exo-miRNAs in Cancer: A Focus on Therapeutic and Diagnostic Applications. Int. J. Mol. Sci..

